# Comparing Social media and Google to detect and predict severe epidemics

**DOI:** 10.1038/s41598-020-61686-9

**Published:** 2020-03-16

**Authors:** Loukas Samaras, Elena García-Barriocanal, Miguel-Angel Sicilia

**Affiliations:** Computer Science Department, Polytechnic Building, University of Alcalá, Ctra. De Barcelona km. 33.6, 28871 Alcalá de Henares, Madrid Spain

**Keywords:** Infectious diseases, Machine learning

## Abstract

Internet technologies have demonstrated their value for the early detection and prediction of epidemics. In diverse cases, electronic surveillance systems can be created by obtaining and analyzing on-line data, complementing other existing monitoring resources. This paper reports the feasibility of building such a system with search engine and social network data. Concretely, this study aims at gathering evidence on which kind of data source leads to better results. Data have been acquired from the Internet by means of a system which gathered real-time data for 23 weeks. Data on influenza in Greece have been collected from Google and Twitter and they have been compared to influenza data from the official authority of Europe. The data were analyzed by using two models: the ARIMA model computed estimations based on weekly sums and a customized approximate model which uses daily sums. Results indicate that influenza was successfully monitored during the test period. Google data show a high Pearson correlation and a relatively low Mean Absolute Percentage Error (*R* = 0.933, *MAPE* = 21.358). Twitter results are slightly better (*R* = 0.943, *MAPE* = 18.742). The alternative model is slightly worse than the ARIMA(X) (*R* = 0.863, *MAPE* = 22.614), but with a higher mean deviation (abs. mean dev: 5.99% vs 4.74%).

## Introduction

The early detection and prediction of the spread of epidemics is an important concern in public health. Traditional systems and techniques mainly use epidemiological data, such as medical data or health log files obtained from doctors and hospitals. However, several studies have recently included non-epidemiological data obtained through the Internet as an alternative, concretely data extracted from search engines or messages interchanged in social media. Results show that this approach may provide a complementary source to detect and predict the volume and spread of unfolding for severe epidemics. These models also include event-based surveillance (EBS) systems and risk modelling^1^ and use available Internet data to detect the evidence of an emerging threat, such as the onset of an epidemic. Some of these tools^2^ combine risk management, signal processing and econometrics to construct a novel method towards a forecast of disease outbreaks in Europe. Also, in some cases, infection dynamics are discussed^3–5^ based on complex networks of exogenous transmitting or environmental factors.

Some researchers further extend this approach to benefit from Internet of Things (IoT) technologies^6^. In addition, recent advances cover the analysis of real-time data or even the sentiment of the data found inside social media^7^, such as Twitter. The latter approach considers social media data as a *word cloud*, from which useful metrics and results can be measured and studied.

Previous works used the Google search engine or social media to track or discuss epidemics, and these two are the main sources for detecting and predicting the spread, the size and the peak of serious diseases. It has been found that nearly two thirds (65.70%) of Internet data fulfill the previous goal originating from the Google Search engine and Twitter^8^. Some previous research use both data sources for health purposes, such as the recent work of Jung *et al*.^9^. Nevertheless, that work does not answer the question about which one is best as a data source: Google or Twitter.

Computer technology offers nowadays many useful tools for mining the Web. In recent years, advanced statistical models have been used to analyze and interpret these data. Auto Regressive Integrated Moving Average model with exogenous (external) variable (ARIMA(X)) is widely used for epidemics, as it has been shown that it can successfully handle Internet data, and also for measurement and forecast^10–12^. Data extraction is possible through writing and executing specific programming code that uses API’s (*Application Programming Interfaces*) through various development frameworks, such as *Pytrends*^13^, *Tweepy*^14^ or *Twython*^15^.

Furthermore, internet data may sometimes show some form of inconsistency, e.g. major rise or descent over time, exaggeration or underestimation of real events (such as epidemics) or repeating presence of the same content, which may significantly affect the detection of epidemics through the Internet.

In this research, we use data from Google Trends^16^ and Twitter to detect influenza in Greece. Data from Google have been obtained through the web application of *Google Trends* and Twitter data through the Twitter *STREAMING API*^17^ and the *Tweepy* library. Two models have been applied for the analysis of data; an ARIMA(X) model based on weekly data and a custom approximate model based on daily data. By creating a real-time information system, we then compared not only Google and Twitter, but also the two sources to each other. Our final objective was to determine the effectiveness of this information system in a low-cost platform, i.e. an application server with inexpensive components.

Based on the above, the specific objectives of this work are:

- To compare and determine which data source is better in tracking epidemics; Google or Twitter, as the current literature provides limited information on this comparison.
- To apply an ARIMA(X) model and compare an approximate model to minimize variances of the prediction values due to any possible inconsistency of the data from Twitter mentioned above.
- To demonstrate that an internet surveillance system may provide with the necessary tools to detect and predict epidemics in real-time with an inexpensive hardware and software configuration.

The rest of this paper is structured as follows: Section 2 describes the methods and tools used for this study, then Section 3 contains the results. Discussion and conclusions are included in the last section. The Appendices contain the description of the used platform and the ARIMA(X) approximation algorithm.

## Methods and Tools

### Research questions

The research plan was designed and executed in three different phases: preparation, data gathering and data analysis. The research was conducted during a nine-month period, from August 1, 2018 until the 30^th^ of June of 2019. The software and hardware preparations lasted about 4 months and a half, the data capturing period was 23 weeks and the analysis phase was about one month.

The plan was designed to answer the following research questions (RQs):

RQ1:Which source can provide more accurate estimation and prediction of the influenza development: Google or Twitter?

RQ2: In which cases can we build and use an approximate model to the ARIMA(X) statistical model with accurate predictions? What are the merits of such as model?

The alternative model mentioned in RQ2 accounts for particularities of the data, concretely: to minimize the effect of repeated messages from Twitter (retweets), or the occurrence of messages with the same or similar content, and to properly adjust and smoothen the prediction curve in cases of a sudden increasing or descending infection impact or when the influenza activity is very low.

### Data

We obtained three data sets for a period of 23 weeks, from December 13, 2018 until May 20, 2019. All sets contain data on influenza in Greece: one of them is from the European Center of Disease Control and Prevention (ECDC). ECDC has established a joint Centre^18^ for Disease Prevention and Control with the World Health Organization (WHO) Regional office. Flu News Europe bulletin includes monitoring data and guidelines on influenza activity in the 50 Member-States of the European Region^19^. The second set came from Google Trends and the third from Twitter. For Google and Twitter, we used as search tokens two terms in the Greek language: *Γρίπη* and *γρίπη*. These are equivalent to the English word *Influenza*. The first keyword is mainly used in the beginning of the sentence, while the second one can be used in the middle. We used both as we tried to have, as much as a big data sample, especially from Twitter.

The Data from Google and ECDC is counted weekly, while Twitter data is real-time and aggregated into daily and also into weekly volumes for comparison purposes. Health data from ECDC corresponds to the *consultation rates* for influenza-like illness (ILI) for Greece, which are presented by the Flu NEWs Europe system. This data is based on nationally organized sentinel networks of primary care physicians, mostly general practitioners (GPs), covering at least 1–5% of the population in their countries and is calculated per 100,000 population. Twitter data are the messages, called *tweets*, which were interchanged between people in Greece, concerning influenza for the period considered. We gathered 18,128 tweets in total during the 23-week period, and we used them all despite that there were repeating or forwarded messages (“retweets”).

### Platform (Hardware and Software)

The hardware included the minimum components to run the designed software. The computer, the operational system and an uninterrupted power supply unit (UPS). The total cost of the hardware was about 375€ and it is shown in the Appendix 1. 628 lines of programming code were written in Python, VB.NET and MS-DOS, while the Windows 10 Pro 64-bit was used as the operational system (OS). The Twitter API, which was used was the *STREAMING API* (instead of the *REST API*^20^), along with the*Tweepy library version.3.6.0*^21^ in *Pyhton 3.5.2 64-bit for Windows*^22^. A part of the software module with the code for the applied calculation algorithm can be found in Appendix 2. The entire system was designed to perform the following tasks:

1. To gather and store the messages from Twitter in plain text files, so that the size of these files can be small, as much as possible. The fields of the files were year, month, day, time, week number, text of the message and the username.
2. To calculate daily and weekly sums of the messages and store them in another text file.
3. To estimate the influenza activity by using the written algorithm, store the results into text files and construct a dynamic real-time graph and tables of measurements on the application window form.
4. To self-test if the connection is broken for some reason and re-establish it to minimize the loss of data.
5. To update current estimation and trend every five seconds.

### Statistical methods

We used the three-parameter Auto Regression Integrated Moving Average model with exogenous (external) variable (ARIMA(X) (p, q, d)) to estimate the influenza development on a weekly basis. This model is constructed by setting the ILI cases as the dependent variable (predicted variable) and Twitter messages as the independent (external) variable. The ARIMA model can be generally thought as discrete time linear equation with noise as shown in equation:

1 $$(\mathop{\sum }\limits_{k=1}^{p}{a}_{k}{L}^{k}){(1-L)}^{d}{X}_{t}=(\mathop{\sum }\limits_{k=1}^{q}{\beta }_{k}{L}^{k}){e}_{t}$$

Where:

p = order of the autoregressive part of the model,

d = order of differencing and

q = order of the moving average part of the model

X_t_ = the predicted value

L = the lag operator

If we assume that **Y**_**t**_ = **(1-L)**^**d**^**X**_**t**_ = **Y**_**t**_ + **X**_**t**_**-**_**1**_ **+ … + X**_**t**_**-**_**n**_, then the model can be expressed as shown in the following equation:

2 $$(\mathop{\sum }\limits_{k=1}^{p}{a}_{k}{L}^{k}){Y}_{t}=(\mathop{\sum }\limits_{k=1}^{q}{\beta }_{k}{L}^{k}){e}_{t}$$

In our forecasting model, we include both time series of tweets and the values of influenza, where Y_t_ stands for the predicted value of influenza cases and X_t_ is the value of tweets.

The above model was compared with the parameters for lag 0 of the Google data, as well as for Twitter data, in order to perform the first comparison. The second comparison was made only for Twitter.

The model that it was finally used was an ARIMA (0,1,0), as shown in the following equation:

3 $${{\bf{Y}}{\boldsymbol{{\prime} }}}_{{\bf{t}}}={{\bf{Y}}}_{{\bf{t}}-{\bf{1}}}+{\bf{0}}{\boldsymbol{.}}106({{\bf{X}}}_{{\bf{t}}}-{{\bf{X}}}_{{\bf{t}}-{\bf{1}}}),\{{\bf{t}}=1,2\ldots 23\}$$

Where **Y′**_**t**_ is the new predicted value of influenza and **X**_t_ the predictor (values from the internet) for every week (t).

We checked the ARIMA(X) model against a customized approximate model. The rationale to do so was that (i) sometimes ARIMA(X) models could be complex enough to be converted into an algorithm and translated into a programming code and (ii) we wish to show that an alternative model could also be used, consisting of specific and consecutive conditions with similar results. This alternative model was simple enough and can be seen in the following set of equations in the following Figure.

**Figure 1:**
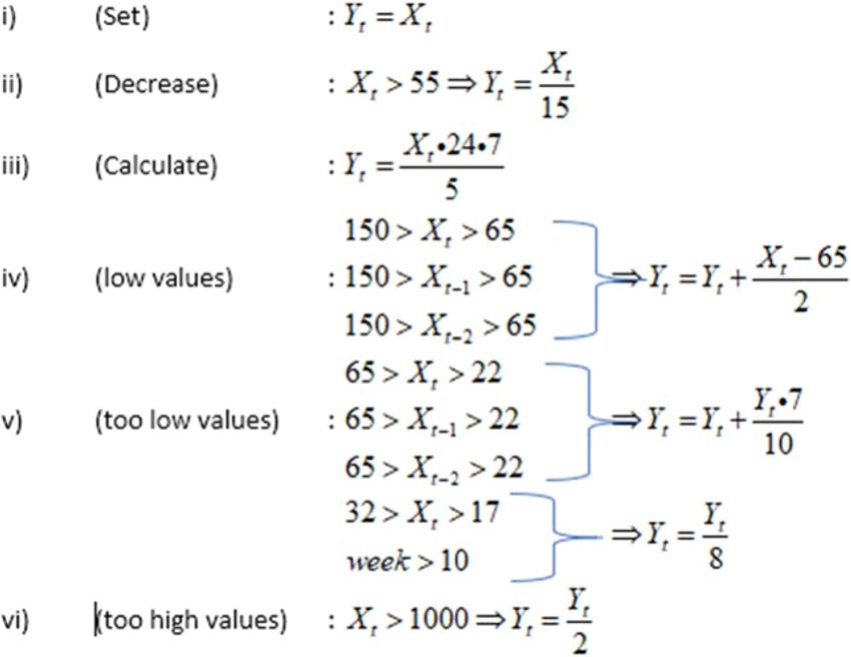
The approximate model algorithm.

As seen in the above Fig. 1, specific adjustments were made to decrease or increase the value of the tweets in special cases. The comparisons were evaluated for precision by using the following criteria:

- Correlation Coefficient (R) and its statistical significance (p-value two-tailed)
- Mean Absolute Percentage Error (MAPE) and
- Mean absolute deviation as percentage of the true influenza cases

As a matter of fact, the presented alternative model is based upon the following three needs:

i) to investigate the capability of tracking the weekly and daily trend based on a daily estimation of influenza, since there is no official daily data,
ii) to minimize the effect of the repeated messages from Twitter (called *Retweets)*, or the occurrence of messages with the same or similar content,
iii) to properly adjust and smoothen the prediction curve in cases of a sudden increasing or descending infection impact or when the influenza activity is very low. This, indeed, happens when the linearity of the data is disturbed, something we have noticed many times when the classic linear models generally fail to deal with this problem.

During the execution of the electronic system and its applications 24/7, we monitored and logged the computer processor (CPU) and memory (RAM) usage and network bandwidth.

## Results: Estimation and Prediction

**Figure 2:**
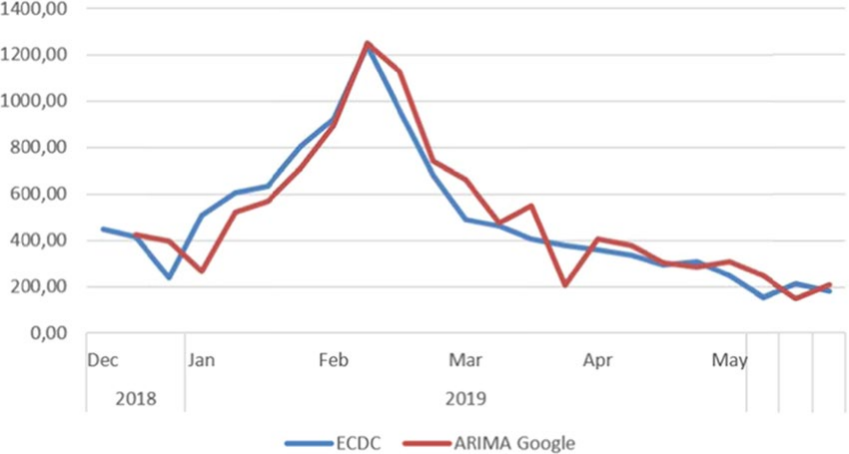
Prediction with Google (ARIMA model).

The first comparison of Google and Twitter reveals that Twitter is slightly better in terms of precision. The values from Google Trends showed a correlation (Pearson R) of 0.933, which is very high and significant at the level of <0.05. Its significance was found to be 0.00000000026, while the observed MAPE was 21.358 and the mean deviation was 4.40%). In the following Fig. 2, we can observe the influenza development of true and predicted cases by using Google data.

**Figure 3:**
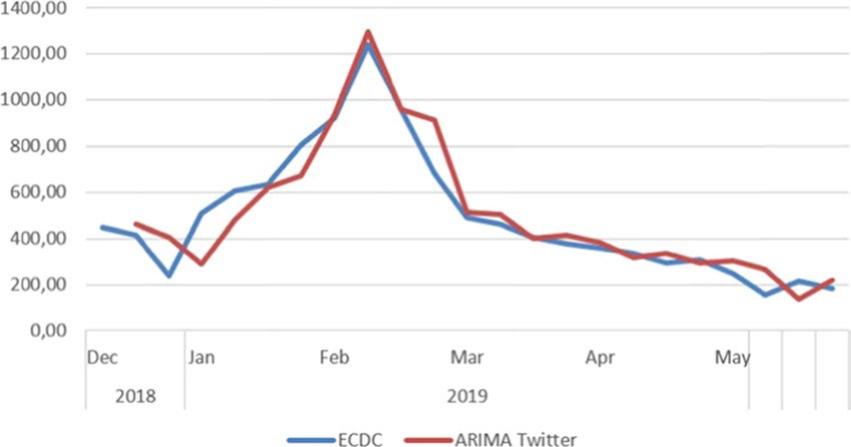
Prediction with Twitter (ARIMA model).

The blue line represents the true influenza cases, measured by the ECDC, while the red line is the predicted values of the ARIMA(X) model based on the Google data. We can observe that the influenza outbreak occurs in the first weeks of February 2019. The model captures this outbreak by calculating it to 1,248.68 cases when, at this time, the actual influenza activity was observed to be 1,178.40. The difference is very small 70.28 cases and it is only 0.56%. The MAPE was measured 21.358, On the other hand, Twitter data show a similar pattern with some small differences from the Google data, as shown in the following Fig. 3.

In the above Fig. 3, we can see that there is also a strong correlation. The correlation coefficient is larger 0.943 at significance level <0.05. However, the peak prediction is slightly higher 1,298.12. The error of the estimate (MAPE) was lower and it was measured 18.742. Finally, the mean deviation was a little bigger (4.74%).

**Figure 4:**
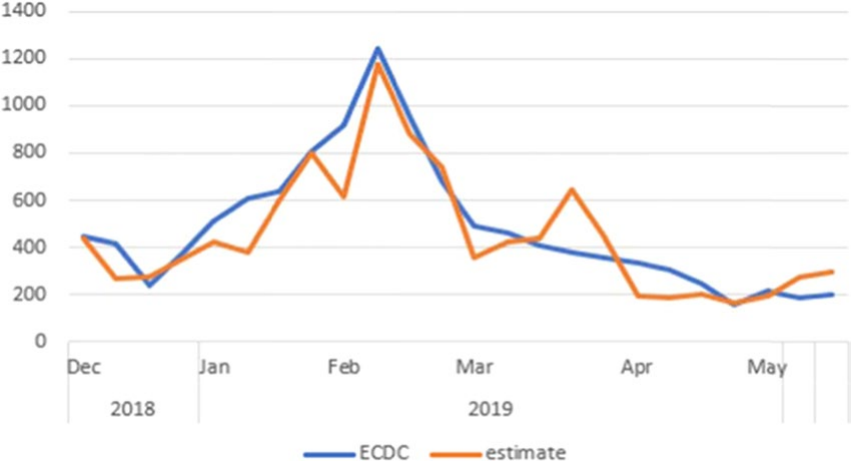
Prediction with Twitter (custom model).

Comparing the results from the ARIMA(X) model and the alternative model, it was found that the custom model was not as good as the ARIMA(X) but could also be considered as reliable. Below, the following graph shows the results (Fig. 4).

The Correlation Coefficient (Pearson R) is lower, but high enough (0.905) at significance level of <0.05 (0.00000011795), while the error (MAPE) was almost the same (22.614) as in the Google model and the absolute mean deviation is higher than ARIMA models, both from Google and Twitter (5.99%). In the following Table 1 we can see all the statistics, calculated for the three models:

**Table 1:** Summary of the results.

n	Statistics	ARIMA Google	ARIMA Twitter	Custom model
1	R	0.933	0.943	0.905
2	sig.	0.00000000026	0.00000000005	0.00000000123
3	MAPE	21.358	18.742	22.614
4	MEAN	503.37	505.03	453.32
5	Mean df	14.73	16.39	−33,56
6	Mean df (%)	4.40%	4.74%	−5.99%
7	Predicted cases	11,522.46	11,558.94	10,426.31
8	Real cases	11,368.68	11,368.68	11,368.68
9	Peak	1,248.68	1,298.12	1,178.40
10	Peak df%	0.56%	4.54%	−5.10%

From the above Table 1 and the figures, we can make some interesting observations:

- All models capture the influenza activity well and estimate the peak at the right time.
- The strongest correlation is shown from the Twitter data (ARIMA(X) Model). All correlations are statistically significant at the level of <0.05
- The smallest error (MAPE) is produced by the Twitter data (ARIMA(X) Model)
- The lowest mean is measured from Twitter (custom model)
- The mean cases of the first two models are a little above 500 cases, slightly higher than the true influenza cases mean (482.1781), while are a little below of the custom model (453.32).
- The peak estimations of the above models are not too much above the real peak (1241.75), except from the custom model, in which is a bit lower (1,178.40).

## Discussion and Conclusions

### Prediction of epidemics

From the above results, it is clearly shown that Internet data can be useful in tracking epidemics. Particularly, Google and Twitter show the potential to estimate and predict influenza, before it is observed in the population. It seems that Twitter has some advantages over Google in our case: it achieves slightly better accuracy, while the monitoring could also be made in smaller time intervals than the official surveillance systems. For instance, European surveillance monitors this epidemic on weekly basis, while, by using Twitter, we are able to monitor the activity and even predict its spread in population daily or within some hours. Google data however is also reliable, but sometimes it was shown^23^ that missed some epidemics, such as the 2009 influenza pandemic through *Google Flu Trends*. In our test case, both data sources were very accurate in estimating the overall development of influenza at the right time and with the right volume. The ARIMA(X) model was proven to be very effective in this process. Different methods have already been proposed for or against ARIMA for health purposes, such the *Holt-Winter* method^24^ or the linear regression methods, e.g. for Ebola^25^. In the latter case, it was shown that a Single Linear Regression (SLR) method resulted in overestimation of the eventual epidemic size, if applied to a real-life data. Scarpino *et al*. (2019) notice that fundamental limits to outbreak prediction are not clear and, and because of this, an integrative approach is needed regarding models for predicting epidemics^26^. In our test case, the results show that epidemics can be certainly predicted by using ARIMA models or even approximate methods. It may though be sometimes very difficult to apply all ARIMA models within the framework of a real-time computer surveillance system. ARIMA Models with many parameters above zero, e.g. ARIMA (2,3,2) require special and time-consuming techniques in order to be realized into a programming code. On the other hand, other custom machine learning models, less accurate, can be easily transferred and integrated into machine code. Our approximate model worked correctly and, despite some of the observed deviations from the ARIMA(X) model, derived interesting results, strong correlation and accuracy in predicting the influenza activity, regarding time and size. Undoubtedly, monitoring of severe epidemics must lead to control and prevention. Influenza has many implications and may result to many deaths, if not prevented and treated in time. In Greece alone, 135 deaths were reported and confirmed during the 2018–2019 season^27^.

### Information systems and data

Internet surveillance systems have the potential to assist the traditional systems of monitoring epidemics. However, during the recent years, there is a discussion on the actual role of them. Particularly, whether they should replace the existing surveillance means or may have a supportive role or should be applied as an extension of the current surveillance tools. Big data, on the other hand, which can be gathered through both traditional and internet surveillance systems, require an effective management. As N. Peek, *et al*.^28^ notice, health and biomedicine data are now abundant, while at the same time too complex to be managed through the traditional methods and practice.

Considering that Twitter users communicate to each other with almost 500 million messages every day and over 200 billion messages every year^29^, we believe that the usability of Twitter as a source of data is obvious. Nevertheless, there is no need for a researcher to capture all tweets or all tweet information with the tools provided from the specific APIs created for this purpose. We can limit this in some hours of day, especially for countries larger than Greece. Of course, data handling should be adequate, and the data itself should be properly used for surveillance of serious epidemics in a way that we can produce reliable results.

Regarding the data, the volume that can be retrieved through the internet is very important. Cholera, for instance, is not a common disease and can be tracked in underdeveloped countries, such as in the regions of West Africa. The severity of influenza is also not the same in all countries for a given period. Nevertheless, when the data size is enough, Twitter has the potential to show the trends regarding the spread of influenza in the countries in which it occurs, in a way that enables estimation and early prediction.

Twitter and Google have some major differences regarding the data that can be extracted. The data from Twitter is plenty as Twitter users communicate to each other frequently each day and furthermore is more detailed than these of Google. This may introduce a significant aspect, specifically when we examine smaller areas and not a vast country such as the US or China. Another difference is the time interval. It is more convenient by using Twitter to allocate more data in smaller segments of time, e.g. less than a day.

### Limitations

Twitter can be used as a very useful data source, specifically when other resources do not provide good results compared to less popular searches. Influenza is considered a disease with a big interest in the public. The analysis of the tweets could be more detailed in terms of time, since we can set the time frame and gather tweets every minute or hour, every 12 or less hours, every day etc. This, of course, requires big databases and extensive analysis, but we believe that the tools are now present and will be further developed as technology advances. In our test models, the size of the data and the produced electronic files were small, and we did not need to localize the tweets for this purpose, since the particularities of the Greek language make them easily identifiable. On the other hand, it could be impossible to track messages from Twitter, based only on the language. For the English term *influenza*, for example, it would be necessary to locate the tweets if we want to study a specific area or a country. This is important since location information (geo-tagging) on Twitter is limited to 0.4–0.5% of the total size of the tweets, while 26% or less of the users report their city-level location. This happens because many users do not want their location to be published on-line. In this case, either the data sample must be big enough or other special techniques must be applied to resolve this issue^30,31^. By contrast, localization of Google search volume can be easily made, both from their web application and through APIs such as Pytrends.

Another aspect is the statistical approach. When a known distribution is identified, it certainly simplifies the analysis process, particularly in cases of low correlation data. However, if the parameters of the distributions are quite different for many different periods, there is a possibility that an alternative method would be probably needed. For example, the use of different simple linear or log linear regressions for separate years or the use of non-parametric mixed models. Generally, it’s the form of data and the desired outcome that defines the most appropriate method to be used, e.g. when we want to maximize the correlation or to achieve better estimation and predictions for the peak of the occurrence of an infectious disease. Nevertheless, in the case of influenza in Greece, the used models both with Google and Twitter provide a good estimation and prediction of the general pattern of the spread, the peak of the disease and the time it occurs.

Nowadays, many different technologies can be used to retrieve real-time tweets, such as the Twitter REST API or the Twitter STREAMING API. REST APIs have limitations of use called *rate limits*^32^. These limits are per user or per application and are divided into 15-minute windows. This means that the catching of the tweets stops, and some time is required to start gathering tweets again. The solution to this problem is to use the streaming API, which can be executed for longer-lived connections with Twitter. This second API has no rate limits, but certain read time-outs occur after some hours of the connection and running the streaming. It is possible though to restart the stream after a while. There are also very popular electronic libraries to be used with the streaming API, such as Twython, Tweepy etc. Unfortunately, it is now too difficult to trace back tweets, even though the Twitter API parameters include the *since* parameter. Despite this fact, it is possible to collect many tweets real-time or from the past tracing the web (https://twitter.com/search-home), but it can be difficult to locate the origin of the tweets. To gain full access to the Twitter API is not allowed by Twitter. There are resellers however^33^, such as DataShift, GNIP Inc. and Topsy, each with different levels of access that can provide large amounts of Twitter data by purchasing them. This access is done by Twitter *Firehose*.

On the other hand, Google data can be found both from the web application of Google or by using specific API’s, such as Pytrends. Google Trends data is an unbiased sample of Google search data and only a percentage of searches are used to compile trends data^34^. This may indicate a small degree of error and the results may be variable to it. The same can be said about the REST API or even more for the streaming API of Twitter. Some API’s, such as Pytrends, may result in non-reliable data when search terms contain non-speaking English words with denotation symbols. With that said, along with the sometimes unpredicting behavior of humans (exaggeration or underestimation reactions to some events), internet data may mislead to wrong results.

### Conclusions

From the current literature, it seems that in many cases it is possible to conduct estimation and prediction patterns for epidemics from internet data. We confirm this assumption and we have further examined Google and Twitter data and found out that both can produce precise and usable estimation of the influenza development. Although the proposed models were applied to influenza, it would be possible that these models or similar ones could produce similar results for other epidemics as well. Nevertheless, this is an assumption that needs to be confirmed. Regarding internet data, Twitter seems to be slightly better, since it provides better prediction for the entire time-series and has some more advantages over Google. The main criterion of assessing the prediction pattern is certainly the accurate prediction of time and volume when an outbreak is noticed. Nevertheless, additional measurements could be applied, and other evaluation statistical criteria can also be used; such as the mean deviation, or the mean absolute percentage error. As far the ARIMA(X) model is concerned, it worked correctly, as well as the approximate alternative model, despite the less accuracy of prediction. In both cases the correlation between real and predicted influenza cases are found to be very strong. This approximate model could be proved very handy when an ARIMA(X) model is too complex to be translated into machine code.

Although Twitter or even Google data require advanced technical skills to be retrieved and analyzed, there is no doubt that it could be useful. Early tracking of epidemics, on the occasions that this can be achieved, could drive to better alerting, controlling and preventing of them. It is also very promising that ad-hoc electronic systems can be implemented with low costs and could certainly be easily managed as supplementary systems in contrast to the traditional monitoring systems. This means that, if the applied models can achieve precise predictions, we don’t really need epidemiological data at all. However, this is partly true, and it is not always validated. This aspect is very important, specifically on some occasions when the forecast is not so good due to the composition or lack of some data or even because of an unexpected reaction to epidemics of people who use search engines or social media.

## Appendices

**The hardware**

**Table:**

#	Component	Model	Cost (US$)	Cost (€)
1	CPU	Intel G3430/3.3GHz dual-core	51.98	45.60
2	GPU	Intel HD Graphics	0.00	0.00
3	RAM	Transcend 3GB/ DDR3-1333MHz	45.00	39.47
4	Motherboard	Gigabyte GA-H81M-D2V	58.71	51.50
5	Monitor	NEC LCD1503M/ 15/1280x1024/75Hz/250Cd^2^	51.30	45.00
6	Wifi Card	Realtek RTL8192EU Wireless LAN 802.11n USB 2.0 Network Adapter	11.69	10.25
7	SSD	Corsair Force LS SSD 128GB	40.93	35.90
8	HDD	WDC 80GB/SATA I/7200rpm	23.37	20.50
9	UPS	1050VA	87.89	77.10
10	Case+PSU	Midi tower+ PSU 350W	51.15	47.50
	**Total**		**425.01**	**372.82**

**The ARIMAX approximation algorithm module**

Imports System

Imports System.IO

Imports System.Text

Module ModuleStats

Public WeekCalculation(5, 12, 31)

Public dataDay(5, 12, 31)

Public dataDay2(5, 12, 31)

Public GraphX(10000) As String

Public Listbox2 As ListBox

Public TimeYear, TimeMonth, TimeWeek, TimeDay, TimeHour, TimeMinute

Public data(100000, 3)

Public StartYear, StartMonth, StartWeek, StartDay, EndYear, EndMonth, EndWeek, EndDay

Public StartHour, EndHour

Public StartMinute, EndMinute

Sub Calculations()

'delete stats

For i = 0 To 2 ' for every year

For k = 1 To 12 ' for every month

For m = 1 To 31 ' for every day

'calculate the week of each day

Dim CurrentDate As Date, FirstDate As Date

'WeekCalculation(i, k, m) = 1 + Int((30.4 * (k - 1) + m) / 7.019) '

If i = 0 And k < 12 Or (i = 0 And k = 12 And m < 13) Then GoTo nextDate ' start writing

If i = EndYear And k > EndMonth Or (i = EndYear And k = EndMonth And m > EndDay) Then GoTo nextDate ' stop writing

If (k = 4 Or k = 6 Or k = 9 Or k = 11) And m = 31 Then GoTo nextDate ' stop writing for months with 30 days

If i + 2018 Mod 4 = 0 Then If k = 2 And m > 29 Then GoTo nextDate ' check February for 29 δαυσ

If k = 2 And m > 28 Then GoTo nextDate ' check February of 28 days

CurrentDate = i + 2018 & "-" & k & "-" & m

FirstDate = i + 2018 & "-01-01" 'first day of the year"

Dim totalDays = 1 + (CurrentDate - FirstDate).TotalDays.ToString ' days from the first day of the year

WeekCalculation(i, k, m) = Math.Ceiling(totalDays / 7) ' calculate week (roundup)

'MsgBox(CurrentDate & "," & ",TotalDays " & totalDays & ",CurrentWeek " & WeekCalculation(i, k, m))

If dataDay(i, k, m) > 0 Then ' show counts >1

dataDay(i, k, m) = 0

End If

nextDate:

Next m

Next k

Next i

' calculate stats

For i = 1 To cRow

TimeYear = Left(data(i, 1), 4) - 2018

TimeMonth = Mid(data(i, 1), 6, 2)

TimeDay = Mid(data(i, 1), 9, 2)

TimeWeek = 1 + Int((30.4 * (TimeMonth - 1) + TimeDay / 7.019))

'TimeWeek = 0

TimeHour = Mid(data(i, 1), 12, 2)

TimeMinute = Mid(data(i, 1), 15, 2)

dataDay(TimeYear, TimeMonth, TimeDay) = dataDay(TimeYear, TimeMonth, TimeDay) + 1

Next i

' Estimate real influenza

For i = 0 To 1 ' for every year

For k = 1 To 12 ' for every month

For m = 1 To 31 ' for every day

' calculation

' adujstement 1

dataDay2(i, k, m) = dataDay(i, k, m)

If dataDay2(i, k, m) > 55 Then dataDay2(i, k, m) = Int(dataDay2(i, k, m) / 15) ' adjustement for influenza

If dataDay2(i, k, m) <> 0 Then

dataDay2(i, k, m) = dataDay2(i, k, m) * 24 * 7 / 5 / 7

End If

' adjustment 2' low values: check continiously big values

If i > 0 And m > 3 Then

If dataDay(i, k, m) > 65 And dataDay(i, k, m - 1) > 65 And dataDay(i, k, m - 2) And

dataDay(i, k, m) < 150 And dataDay(i, k, m - 1) < 150 And dataDay(i, k, m - 2) < 150 And

dataDay(i, k, m) IsNot Nothing Then ' check <65tweets<150

For x = m - 2 To m

If dataDay(i, k, x) > 65 Then dataDay2(i, k, x) = dataDay2(i, k, x) + 0.5 * (dataDay(i, k, x) - 65)

'If dataDay2(i, k, x) < 0 Then dataDay2(i, k, x) = Math.Abs(dataDay2(i, k, x))

Next x

End If

End If

' adjustment 2b' too low values: check continiously big values

If dataDay(i, k, m) > 22 And dataDay(i, k, m) < 65 Then

If dataDay(i, k, m) < 65 And dataDay(i, k, m - 1) < 65 And

dataDay(i, k, m) > 22 And dataDay(i, k, m - 1) > 22 And

dataDay(i, k, m) IsNot Nothing Then ' check <65tweets<150

For x = m - 2 To m

dataDay2(i, k, x) = dataDay(i, k, x) * 0.7

'If dataDay2(i, k, x) < 0 Then dataDay2(i, k, x) = Math.Abs(dataDay2(i, k, x))

Next x

End If

End If

' too low values after week 10a

If WeekCalculation(i, k, m) > 10 And WeekCalculation(i, k, m) < 52 And dataDay(i, k, m) > 17 And dataDay(i, k, m) < 32 Then dataDay2(i, k, m) = dataDay2(i, k, m) / 8

' adjustment 3: very high values

If dataDay(i, k, m) > 1000 Then dataDay2(i, k, m) = dataDay2(i, k, m) / 2 ' adjustment3 too high

Next m

Next k

Next i

'time of last entry

TimeMinute = Mid(data(cRow, 1), 15, 2) ' last minute recording

TimeStamp = data(cRow, 1)

End Sub

Sub CalculationTotal()

Dim OutputMonth, OutputDay, OutputWeek

' create file

Dim path As String = "D:\Python\data\StatsDay.txt"

If Not File.Exists(path) Then

' Create a file to write to.

Using sw As StreamWriter = File.CreateText(path)

End Using

End If

' open files to write

Dim objWriter2 As New System.IO.StreamWriter(path)

' calculate sums and write

objWriter2.WriteLine("yyyy-mm-dd" & Chr(9) & "week" & Chr(9) & "tweets" & Chr(9) & "estimate") ' write headers

'MsgBox(dataDay2(1, 1, 1))

For i = StartYear To EndYear ' for every year

For k = 1 To 12 ' for every month

For m = 1 To 31 ' for every day

If k < 10 Then OutputMonth = "0" & k Else OutputMonth = k 'If dataDay(i, k, m) Is Nothing Then dataDay(i, k, m) = 0

If m < 10 Then OutputDay = "0" & m Else OutputDay = m

If WeekCalculation(i, k, m) < 10 Then OutputWeek = "0" & WeekCalculation(i, k, m) Else OutputWeek = WeekCalculation(i, k, m)

' check start

If i = 0 And k < 12 Or (i = 0 And k = 12 And m < 13) Then GoTo Notwrite ' start writing

If i = EndYear And k > EndMonth Or (i = EndYear And k = EndMonth And m > EndDay) Then GoTo Notwrite ' stop writing

If (k = 4 Or k = 6 Or k = 9 Or k = 11) And m = 31 Then GoTo Notwrite ' stop writing for months with 30 days

If i + 2018 Mod 4 = 0 Then If k = 2 And m > 29 Then GoTo Notwrite ' check February for 29 δαυσ

If k = 2 And m > 28 Then GoTo Notwrite ' check February of 28 days

objWriter2.WriteLine(i + 2018 & "-" & OutputMonth & "-" & OutputDay & Chr(9) & OutputWeek & Chr(9) & dataDay(i, k, m) & Chr(9) & dataDay2(i, k, m)) ' store tweets stats

Notwrite:

Next m

Next k

Next i

objWriter2.Close()

End Sub

End Module dataDay(i, k, m) IsNot Nothing Then ' check <65tweets<150

For x = m - 2 To m

dataDay2(i, k, x) = dataDay(i, k, x) * 0.7

Next x

End If

End If

' adjuestemnt 3: very high values

If dataDay(i, k, m) > 1000 Then dataDay2(i, k, m) = dataDay2(i, k, m) / 2 ' adjustment3 too high

Next m

Next k

Next i

'time of last entry

TimeMinute = Mid(data(cRow, 1), 15, 2) ' last minute recording

TimeStamp = data(cRow, 1)

End Sub

## References

[1] Rees, E. E. *et al* Early detection and prediction of infectious disease outbreaks (2019), *CCDR* 45 5), May 2, 2019, ISSN**:** 1481–8531 (2019). 10.14745/ccdr.v45i05a02 PMC6587687 31285702

[2] Hassani H, Reza Yeganegib M, Sirimal Silva E, Ghods F (2019). Risk management, signal processing and econometrics: A new tool for forecasting the risk of disease outbreaks. Journal of Theoretical Biology.

[3] Li L (2019). Analysis of transmission dynamics for Zika virus on networks. Applied Mathematics and Computation 2019.

[4] Yi, W. & JinDe, C. Final size of network epidemic models: Properties and connections, S*cience China Information Sciences*, 10.1007/s11432-019-2656-2 (2019).

[5] Yi W, Jinde C, Gang H (2019). Further dynamic analysis for a network sexually transmitted disease model with birth and death. Applied Mathematics and Computation.

[6] Kaushalya, S. A. D. S., Kulawansa K. A. D. T. & Firdhous M. F. M. Internet of Things for Epidemic Detection: A Critical Review. In: Bhatia, S., Tiwari, S., Mishra, K. & Trivedi, M. (eds). Advances in Computer Communication and Computational Sciences. Advances in Intelligent Systems and Computing, vol 924. *Springer*, Singapore, 10.1007/978-981-13-6861-5_42 (2019).

[7] Sanjiv, K., Bhatia, S. K., Mishra, K. K. & Trivedi, M. C. Advances in Computer Communication and Computational Sciences: Proceedings of IC4S 2018 (Advances in Intelligent Systems and Computing) 1st Edition, Kaushalya, S. A. D. S. *et al*., Springer pp. 480–488, ISBN-13: 978-9811368608, ISBN-10: 9811368600 (2019).

[8] Samaras, L., Garcia-Barriocanal, E & Sicilia, M. A. Syndromic surveillance models using Web data: a systematic review, Book by Lytras M., Sarirete A., *Innovation in Health Informatics, 1st Edition, A Smart Healthcare Primer*, Chapter 2, p.39–77, Elsevier Science Publishing Co Inc., Imprint by Academic Press Inc 13.11.2019, ISBN: 9780128190432, ISBN10: 0128190434, ISBN13: 9780128190432, 10.1016/B978-0-12-819043-2.00002-2 (2019).

[9] Jung, J., Uejio, C. K., Duclos, C. & Jordan, M. Using Web Data to Improve Surveillance for Heat Sensitive Health Outcomes Environmental Health. Environmental Health 18, Article number: 59. 10.1186/s12940-019-0499-x (2019). 10.1186/s12940-019-0499-x PMC6615306 31287016

[10] Kang L (2019). Using Baidu Search Engine to Monitor AIDS Epidemics Inform for Targeted intervention of HIV/AIDS in China. Scientific Reports.

[11] Jing, Q. L, Cheng, Q., Marshall, J. M., Hu, W. B. Imported cases and minimum temperature drive dengue transmission in Guangzhou, China: evidence from ARIMAX model, *Epidemiology & Infection* **146**(10), 10.1017/S0950268818001176 (2018). 10.1017/S0950268818001176 PMC9134281 29781412

[12] Chadsuthi, S., Iamsirithaworn, S., Triampo, W. & Modchang, C. Modeling Seasonal Influenza Transmission and Its Association with Climate Factors in Thailand Using Time-Series and ARIMAX Analyses. *Computational and Mathematical Methods in Medicine* 2015, Article ID 436495. 10.1155/2015/436495 (2015). 10.1155/2015/436495 PMC4667155 26664492

[13] General Mills. Pytrends, https://github.com/GeneralMills/pytrends (2019).

[14] Tweepy, https://www.tweepy.org/ (2019).

[15] Twython, https://twython.readthedocs.io/en/latest/ (2019).

[16] Google Trends, https://trends.google.com/trends (2019).

[17] Twitter Development Documentation. Streaming APIs, https://dev.twitter.com/streaming/overview (2018).

[18] The Joint European Centre for Disease Prevention and Control (ECDC)–WHO Regional office, https://flunewseurope.org/System (2019).

[19] Flu News Europe, https://flunewseurope.org/CountryData?country=EL (2019).

[20] Twitter REST-API, Rules and Policies, https://help.twitter.com/en/rules-and-policies/twitter-apihttps://www.w3resource.com/API/twitter-rest-api/ (2019).

[21] Tweep. Documentation, https://tweepy.readthedocs.io/en/latest/ (2019).

[22] Python, https://www.python.org/downloads/release/python-352/ (2019).

[23] Olson, D. R., Konty, K. J., Paladini, M., Viboud, C. & Simonsen, L. Reassessing Google Flu Trends data for detection of seasonal and pandemic influenza: a comparative epidemiological study at three geographic scales. *PLoS Comput Biol* Oct 17, **9**(10), e1003256. 10.1371/journal.pcbi.1003256 (2013). 10.1371/journal.pcbi.1003256 PMC3798275 24146603

[24] Tanyavutti, A. & Tanlamai, U. ARIMAX versus Holt Winter Methods: The Case of Blood Demand Prediction in Thailand, International Journal of Environmental & Science Education, **13**(6), 519–525, e-ISSN: 1306–3065 (2018).

[25] Verkerk, L. Thesis advisor: Prof. Dr. Wallinga J., Second thesis advisor: Prof. Dr. Putter H. Forecasting Infectious Disease Epidemics, Master Thesis, University of Leiden, https://www.universiteitleiden.nl/binaries/content/assets/science/mi/scripties/statscience/2017-2018/2018_06_29_masterthesis_verkerk.pdf (2018).

[26] Scarpino, S. V. & Petri, G. On the predictability of infectious disease outbreaks, *Nature Communications*, **10**(1), 10.1038/s41467-019-08616-0 (2019). 10.1038/s41467-019-08616-0 PMC6385200 30796206

[27] Greek National Health Organization, Influenza Weekly report (week 14/2019), 11.04.2019, https://keelpno.gr/wp-content/uploads/2019/01/14.2019-Flu-Week.pdf (2019).

[28] Peek N, Holmes JH, Sun J (2014). Technical challenges for big data in biomedicine and health: Data sources, infrastructure, and analytics. Yearbook of Medical Informatics.

[29] Internet Live Stats. Twitter user statistics, http://www.internetlivestats.com/twitter-statistics (2019).

[30] Bounding Box, http://boundingbox.klokantech.com/ (2019).

[31] Mahmud, J., Nichols, J. & Drews, C. Home location identification of twitter users. CoRR abs/1403.2345:2014, https://arxiv.org/pdf/1403.2345 (2019).

[32] Twitter Development Documentation. REST API Rate Limits. https://dev.twitter.com/rest/public/rate-limiting (2019).

[33] Kumar, S., Morstatter, F. & Liu, H. Twitter Data Analytics, Springer, New York, NY, USA 2013, http://tweettracker.fulton.asu.edu/tda/TwitterDataAnalytics.pdf (2019).

[34] Google Trends. Where Trends data comes from, available from, https://support.google.com/trends/answer/4365533?hl=en&ref_topic=6248052 (2019).

